# Urinary Dysfunction in Myasthenic Syndromes: A Scoping Review of Clinical Features and Treatment‐Related Associations

**DOI:** 10.1002/mus.70337

**Published:** 2026-07-07

**Authors:** Julia M. Augustin, Lillith Horstkorte, Hellen Schmidt, Blanca G. Sánchez Navarro, Avan Kader, Inga Koneczny, Holly M. Nelson, Pratishtha P. Wadnerkar, Rafael Meleka Hanna, Ayşe N. Özdağ Acarlı, Piraye Oflazer, Atay Vural, Franz Blaes, Miriam L. Fichtner

**Affiliations:** ^1^ Department of Neurology with Experimental Neurology Charité Universitätsmedizin Berlin Berlin Germany; ^2^ Technical University of Munich Department of Diagnostic and Interventional Radiology Munich Germany; ^3^ Division of Neuropathology and Neurochemistry, Department of Neurology Medical University of Vienna Vienna Austria; ^4^ School of Medicine, Department of Neurology Koç University Istanbul Türkiye; ^5^ Research Center for Translational Medicine (KUTTAM) Koç University Istanbul Türkiye; ^6^ Department of Neurology Klinikum Oberberg Gummersbach Germany

**Keywords:** congenital myasthenic syndromes, Lambert‐Eaton myasthenic syndrome, myasthenia gravis, myasthenic syndromes, urinary dysfunction

## Abstract

Urinary dysfunction has been reported in association with myasthenic syndromes, including myasthenia gravis (MG), Lambert–Eaton myasthenic syndrome (LEMS), and congenital myasthenic syndromes (CMS), but evidence regarding its prevalence, clinical impact, pathophysiology, and management remains limited. This scoping review synthesizes the available evidence on urinary symptoms, diagnostic approaches, pathophysiological features, treatment‐related associations, and sex‐specific findings in these disorders. A literature search was conducted in PubMed, LIVIVO, Epistemonikos, and the Cochrane Library in accordance with PRISMA guidelines. Of 774 records identified, eight studies met the inclusion criteria, comprising two case reports, three case–control studies, one prospective study, and two retrospective observational studies. Seven studies addressed MG and one addressed LEMS; no eligible studies were identified for CMS. Reported urinary symptoms were predominantly storage lower urinary tract symptoms (LUTS), including urinary incontinence, urgency, nocturia, and increased frequency, whereas voiding and bladder‐emptying abnormalities were described less consistently. Diagnostic approaches were heterogeneous and included questionnaires, clinical examination, urodynamic testing, imaging, and autonomic assessments. Proposed mechanisms remain uncertain, with limited evidence supporting contributions from pelvic floor weakness, autonomic dysfunction, and cholinesterase inhibitor exposure. Treatment‐related associations were largely observational and most consistently implicated pyridostigmine in symptom worsening, while evidence for specific treatment of urinary symptoms was sparse. Sex‐specific analyses were limited and did not identify consistent sex‐related patterns. Current evidence indicates that urinary symptoms have been reported in myasthenic syndromes and have been associated with reduced quality of life in the included studies, but the available data remain insufficient to define prevalence, mechanisms, or optimal management.

**Abbreviations**
AChR: acetylcholine receptor
CASP: Critical Appraisal Skills Programme
ChEI: cholinesterase inhibitor
CMS: congenital myasthenic syndromes
ECG: electrocardiogram
HC: healthy control
HRV: heart rate variability
ICIQ‐OAB: International Consultation on Incontinence Questionnaire Overactive Bladder
ICIQ‐SF: International Consultation on Incontinence Questionnaire‐Short Form
IVIg: intravenous immunoglobulin
LEMS: Lambert‐Eaton myasthenic syndrome
LOMG: late‐onset myasthenia gravis
LRP4: low‐density lipoprotein receptor‐related protein 4
LUTS: lower urinary tract symptoms
MG: myasthenia gravis
MG‐ADL: Myasthenia Gravis Activities of Daily Living
MGFA: Myasthenia Gravis Foundation of America
MG‐QOL15: 15‐item Myasthenia Gravis Quality of Life scale
MuSK: muscle‐specific kinase
NMJ: neuromuscular junction
OAB: overactive bladder
OABSS: Overactive Bladder Symptom Score
PRISMA: Preferred Reporting Items for Systematic Reviews and Meta‐Analyses
QMG: Quantitative Myasthenia Gravis score
QoL: quality of life
ROBINS‐I: Risk Of Bias In Non‐randomized Studies—of Interventions
RTX: rituximab
SNMG: seronegative myasthenia gravis
VGCC: voltage‐gated calcium channel

## 1 Introduction

Myasthenic syndromes are a group of rare neuromuscular junction (NMJ) disorders characterized by fluctuating, fatigable skeletal muscle weakness. These include autoimmune disorders such as myasthenia gravis (MG) [1, 2, 3] and Lambert–Eaton myasthenic syndrome (LEMS) [4], as well as congenital myasthenic syndromes (CMS) caused by pathogenic variants in NMJ‐associated genes [5, 6], all resulting in impaired neuromuscular synaptic transmission. Non‐motor manifestations remain incompletely characterized. Although autonomic symptoms have been described in LEMS [7, 8], urinary dysfunction in myasthenic syndromes has not been systematically studied, and the available evidence on lower urinary tract symptoms (LUTS) remains insufficient to define their prevalence, severity, or impact on quality of life.

Urinary dysfunction of the lower urinary tract encompasses disturbances in urine storage and voiding. LUTS represent the clinical manifestation of such dysfunction and include incontinence, urgency, frequency, nocturia, and frequency [9]. These symptoms may arise from a range of underlying conditions, including urinary tract infection, bladder outlet obstruction, urinary retention, and neurogenic bladder disorders [10]. Bladder function depends on coordinated autonomic control of detrusor activity and urethral sphincter function, and disruption of this control may contribute to urinary dysfunction [10]. LUTS are highly prevalent in the general population, affecting more than half of adults worldwide, with increasing prevalence with age and, for moderate‐to‐severe symptoms, male sex [11, 12, 13]. Given the central role of neural control in bladder function, LUTS have been increasingly studied in neurological disorders [10, 14, 15, 16].

This scoping review synthesizes the available evidence on urinary dysfunction in myasthenic syndromes, with a focus on MG, LEMS, and CMS. The aim is to map reported urinary symptoms, diagnostic approaches, and treatment‐related associations, as well as to summarize reported pathophysiological features. Given the limited and heterogeneous nature of the available data, the objective is not to derive prevalence estimates but to identify patterns and gaps in the literature. Specifically, this review addresses the following questions: (1) what urinary symptoms have been described in patients with myasthenic syndromes; (2) what diagnostic methods have been used to assess urinary dysfunction; and (3) what associations with therapeutic interventions have been reported.

## 2 Methods

### 2.1 Study Design

Given the heterogeneity of study designs, rarity of urinary manifestations, and absence of standardized outcome measures, this review was conducted as a scoping review to map the extent, nature, and characteristics of the available evidence on urinary dysfunction in MG and related disorders. The database search was conducted on April 05 2025 by two independent reviewers; screening and eligibility assessment were performed between April and August 2025 in accordance with PRISMA (Preferred Reporting Items for Systematic reviews and Meta‐Analyses) guidelines.

### 2.2 Search Strategy

Searches were performed in PubMed, Cochrane Library, Epistemonikos, and LIVIVO by two independent reviewers. Search terms included combinations of the following terms: “Myasthenia Gravis”, “Lambert‐Eaton Myasthenic Syndrome”, “Myasthenic Syndromes, Congenital”, “neuromuscular disease*”, “myasthenia”, “myasthenic syndrome*”, “Lambert‐Eaton syndrome”, “congenital myasthenic syndrome”, “urinary bladder disease”, “urinary bladder”, “urinary bladder, neurogenic”, “lower urinary tract symptoms”, “urinary incontinence”, “bladder dysfunction*”, bladder disorder*”, “neurogenic bladder”, “urinary dysfunction*”, “urinary symptom*”, “incontinence”, “voiding”, “urgency”, “nocturia”, “pollakiuria”, “pollakisuria”, “LUTS”, “overactive bladder”, “OAB”, “dysuria”, “urinary retention”, “detrusor”, “bladder atony”, “residual urine”, “sphincter hypotonia”, “autonomic dysfunction”, “autonomic symptom*”, “dysautonomia”. In PubMed/MEDLINE, terms were entered as MeSH terms and keywords, in Cochrane Library and LIVIVO databases as keywords only, and in the Epistemonikos database as keywords in title and abstract. The Boolean operator AND was applied to separate block A, which contained the underlying diseases MG, LEMS, and CMS, from block B, which contained all autonomic and urinary symptoms. The Boolean operator OR was applied to separate the elements from each block. LIVIVO results were additionally filtered by Language = English and German; Subject = Medicine, Health, and results from the MEDLINE database were excluded. Search results were exported as CSV files, imported into R Studio, merged into a single dataset using the dplyr package, and screened for duplicates based on author lists.

### 2.3 Study Selection and Eligibility Criteria

The filtered records were subsequently screened by two independent reviewers for the inclusion criteria, topic relevance, publication type (excluding reviews), full‐text availability, language (English or German), and ethical requirements according to the Declaration of Helsinki, including informed consent and ethical approval where applicable. Case reports were included to capture rare and under‐recognized urinary manifestations and to support hypothesis generation in an area with limited higher‐level evidence. Records on species other than humans were excluded. Final study inclusion was determined by three independent reviewers using the appropriate CASP (Critical Appraisal Skills Programme) checklists for each study design.

### 2.4 Data Extraction and Risk of Bias

In addition, risk of bias in the included non‐randomized studies was assessed using ROBINS‐I (Figure S1). For each included study, we extracted information on sample size, age, sex, specific myasthenic diagnosis, antibody status, study design, interventions, bladder symptoms, proposed pathophysiological mechanisms, diagnostic workup, and reported treatments (Table 1). The review protocol was not registered in a public database.

**TABLE 1:** Characteristics of included studies.

Publication	Disease and antibody status	Population	Methods	Symptoms	Treatment	Results and diagnosis
Case reports						
Kwegyir‐Aggrey et al. [17]	MuSK+ MG	*n* = 1, female, 71 years	Observation of symptoms	Incontinence, worsening while walking and standing	Incontinence pads Pyridostigmine Pelvic floor exercise Immuno‐suppressive treatment and RTX	Worsening symptoms upon pyridostigmine treatment No improvement of symptoms upon pelvic floor exercise Slight improvement of symptoms upon immunosuppressive treatment Improvement of symptoms upon RTX treatment
Pereira et al. [18]	AChR+ MG and Thymoma	*n* = 1, female, 68 years	Objective examination Urodynamic studies	Thyroidectomy in 2015 Bilateral palpebral ptosis with fatigability Stress incontinence Continuous leakage Decreased quality of life	Incontinence pads No improvement upon functional rehabilitation of pelvic floor Azathioprine Sertraline Prednisone	Positive stress test with empty bladder Reduced muscle strength Decreased anal tone Decreased sphincter contractility Intrinsic sphincter dysfunction Underactive detrusor
Case control studies						
Tateno et al. [19]	AChR+ MG	MG group *n* = 21, 15 female/6 male, 22–73 years (mean 47) HC group *n* = 235, 120 female/115 male, 30–69 years (mean 48)	LUTS questionnaire	MGFA 2–3 Weakness and fatigue in ocular and limb muscles 33% Urgency (17% in control group) 43% incontinence (21% in control group) 24% nocturia (6% in control group)	Thymectomy Prednisolone 5–20 mg/day Pyridostigmine 60–180 mg/day Tacrolimus 3 mg/day Steroid pulse therapy with plasmapheresis Milnacipran (*n* = 1) for urinary symptoms	Reduced QoL in MG compared to healthy control group LUTS appeared after onset of motor disorders and progressed LUTS more common upon ChEI (*n* = 16, 78%) and corticosteroid treatment (*n* = 17, 82%), not significant
Akan et al. [20]	AChR+ and SNMG	MG group *n* = 36, 18 female/18 male, mean age 54.1 years HC group *n* = 29, 15 female/14 male, mean age 50.2 years	ICIQ‐SF OABSS Uroflowmetry Ultrasonography	Generalized MG symptoms 81% nocturia 61% incontinence 47% urgency 25% residual urine 8% voiding	Thymectomy No more information about other therapeutic strategies	Significant more OABSS (*p* = 0.008) Duration of LUTS longer in LOMG (*p* = 0.029) Nocturia more common in LOMG (*p* = 0.023) Duration of LUTS longer in AChR‐MG (*p* = 0.003) Overactive bladder
Donskov et al. [21]	MuSK+ and SNMG	MG group *n* = 83, 42 female/41 male, mean age 58 years HC group *n* = 50, 21 female/29 male, mean age 59 years	QMG MG composite scale MG‐ADL MG‐QOL15 ICIQ‐OAB	Polyuria Nocturia Urgency Stress incontinence	Pyridostigmine Pyridostigmine + immuno‐suppressants Immuno‐suppressants	Patients receiving high (> 300 mg/d) pyridostigmine doses have higher OAB score QMG correlates weakly with OAB (Not significant: *p* = 0.03) Higher total OAB score (*p* < 0.005)
Retrospective observational study						
Mantegazza et al. [22]	LEMS P/Q‐type VGCC+	*n* = 69, 32 female/36 male, mean age 61.5 years	QMG Electro‐physiology Muscle strength assessment Ataxia assessment Electrocardio‐graphy Pulmonary function assess‐ment Questionnaires	Reduced reflexes Reduced muscle strength Abnormal bladder function (24%) Constipation Dry mouth Abnormal pupil reflex Sexual disorders	Firdapse Pyridostigmine Prednisone 3,4‐DAP Azathioprine IVIg therapy Mycophenolate mofetil Cyclophosphamide Cyclosporine	Autonomic nervous dysfunction
Remijn‐Nelissen, Verschuuren & Tannemaat [23]	AChR+ MG MuSK+ MG LRP4+ MG SNMG	*n* = 410 220 female/190 male Mean age 61.1 years	Questionnaire	Urinary urgency (approximately 35%) Flatulence Diarrhea	Distigmine Corticosteroids Azathioprine Rituximab Intravenous Ig Plasmapheresis	Incontinence higher in patients treated with pyridostigmine Lower doses or discontinuing pyridostigmine decreases incontinence
Prospective study						
Nikolic et al. [24]	AChR+ and MuSK+ MG Thymoma	AChR+ with thymoma group *n* = 27, 15 female/12 male, mean age 52.3 years HC group *n* = 23, 12 female/11 male, mean age 50.7 years AChR+ without thymoma group *n* = 25, 23 female/2 male, mean age 40.8 years HC group *n* = 14, 13 female/1 male, mean age 43.2 years MuSK+ group *n* = 23, 18 female/5 male, mean age 47.3 years HC group *n* = 18, 14 female/4 male, mean age 47.2 years	Antibody analysis Cardiovascular reflex RR‐variability Handgrip test HRV after standing up Deep breathing Valsalva 24h‐ECG Questionnaires	AChR+ with thymoma = 17/21 (81%) AChR+ without thymoma = 6/23 (26%) MuSK+ = 5/23 (22%)	Pyridostigmine Prednisone, Azathioprine, Cyclosporine	Urinary and sexual dysfunctions are more frequent in MG Other autonomic symptoms common

Abbreviations: 3,4‐DAP: 3,4‐Diaminopyridine; AChR +/−: acetylcholine receptor‐autoantibodies positive or negative; ChEI: acetylcholinesterase inhibitor; CMS: congenital myasthenic syndromes; ECG: electrocardiogram; EOMG: early‐onset myasthenia gravis; HC: Healthy control; HRV: heart rate variability; ICIQ‐OAB: International Consultation on Incontinence Questionnaire Overactive Bladder Module; ICIQ‐SF: International Consultation on Incontinence‐Short Form; IVIg: intravenous immunoglobulin; LEMS: Lambert‐Eaton myasthenic syndrome; LOMG: late‐onset myasthenia gravis; LUTS: lower urinary tract symptoms; MG: myasthenia gravis; MG‐ADL: Activities of Daily Living scoring system for myasthenia gravis patients; MG‐QOL15: 15‐question assessment of quality of life for myasthenia gravis patients; MuSK+: muscle‐specific kinase‐autoantibodies positive; *n*: number of participants; OAB: overactive bladder; OABSS: overactive bladder symptom score; QMG: quantitative myasthenia gravis score; QoL: quality of life; RTX: rituximab; SN: seronegative.

## 3 Results

### 3.1 Study Selection

**FIGURE 1:**
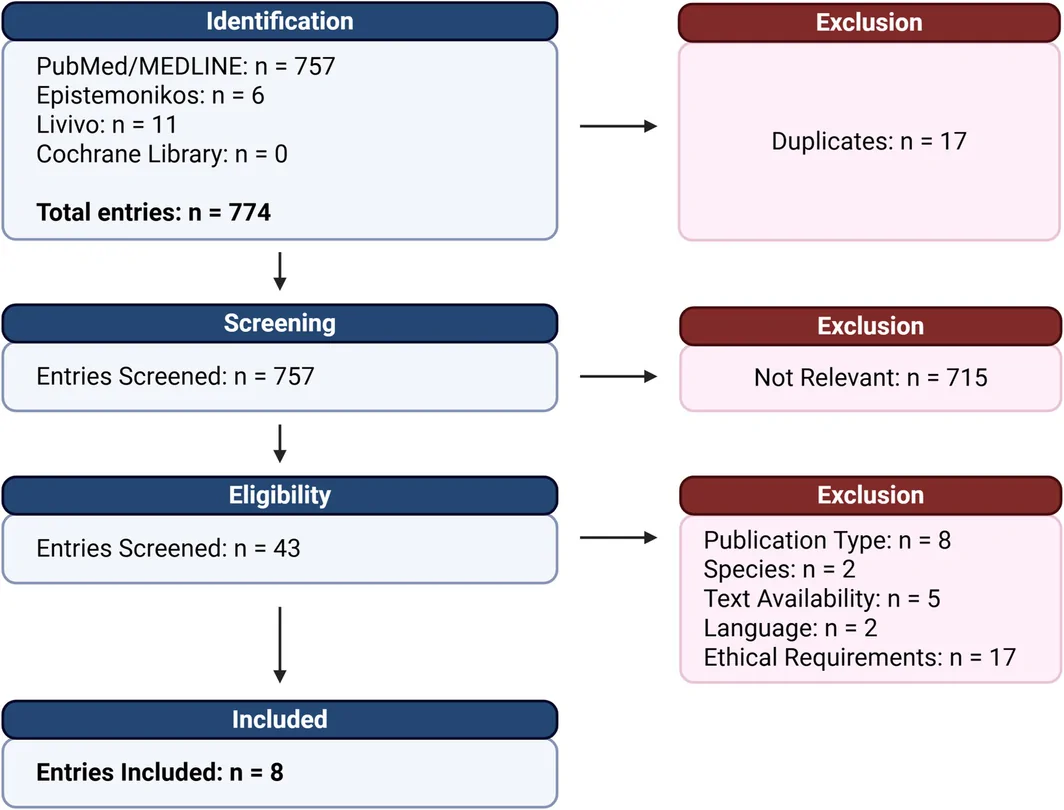
PRISMA flow diagram of study selection. Records identified through searches of PubMed, LIVIVO, Epistemonikos, and the Cochrane Library between April 05 and August 28, 2025 were screened for eligibility. After removal of duplicates and exclusion of non‐relevant or ineligible records, eight studies were included in the final scoping review.

The database search yielded a total of 774 records: PubMed (*n* = 757), Epistemonikos (*n* = 6), LIVIVO (*n* = 11), and Cochrane Library (*n* = 0). After exclusions, a total of eight records were included in the analysis (Figure 1, Table 1). These include two retrospective observational studies on LEMS and MG on autonomic symptoms, including urinary dysfunction [22, 23], three case–control studies in MG [19, 20, 21], one prospective study evaluating autonomic features, including bladder symptoms, in MG with thymoma [24] and two case reports describing urinary dysfunctions in MG [17, 18]. No studies meeting the inclusion criteria addressed urinary dysfunction in CMS.

### 3.2 Urinary Symptom Assessment

Urinary symptoms were assessed using a combination of patient‐reported questionnaires [17, 18, 19, 20, 21, 23], clinical examinations [17, 18, 19, 20], urodynamic testing [18], and imaging‐based evaluations [20] (Table 1). Symptom burden was most commonly evaluated with validated questionnaires, including LUTS questionnaires [19, 20, 21], the International Consultation on Incontinence Questionnaire‐Short Form (ICIQ‐SF) [20], the Overactive Bladder Symptom Score (OABSS) [20], and the International Consultation on Incontinence Questionnaire Overactive Bladder (ICIQ‐OAB) [21]. Objective assessments included focused neurological and pelvic examinations [17, 18, 19, 20], urodynamic studies [18], uroflowmetry [20], and ultrasonography to assess post‐void residual urine [20]. In one study, autonomic function testing and disease‐specific clinical scales were additionally used to explore associations between urinary symptoms, neuromuscular disease severity, and autonomic dysfunction [24].

### 3.3 Urinary Symptoms, Pathophysiology, and Diagnostic Findings

**FIGURE 2:**
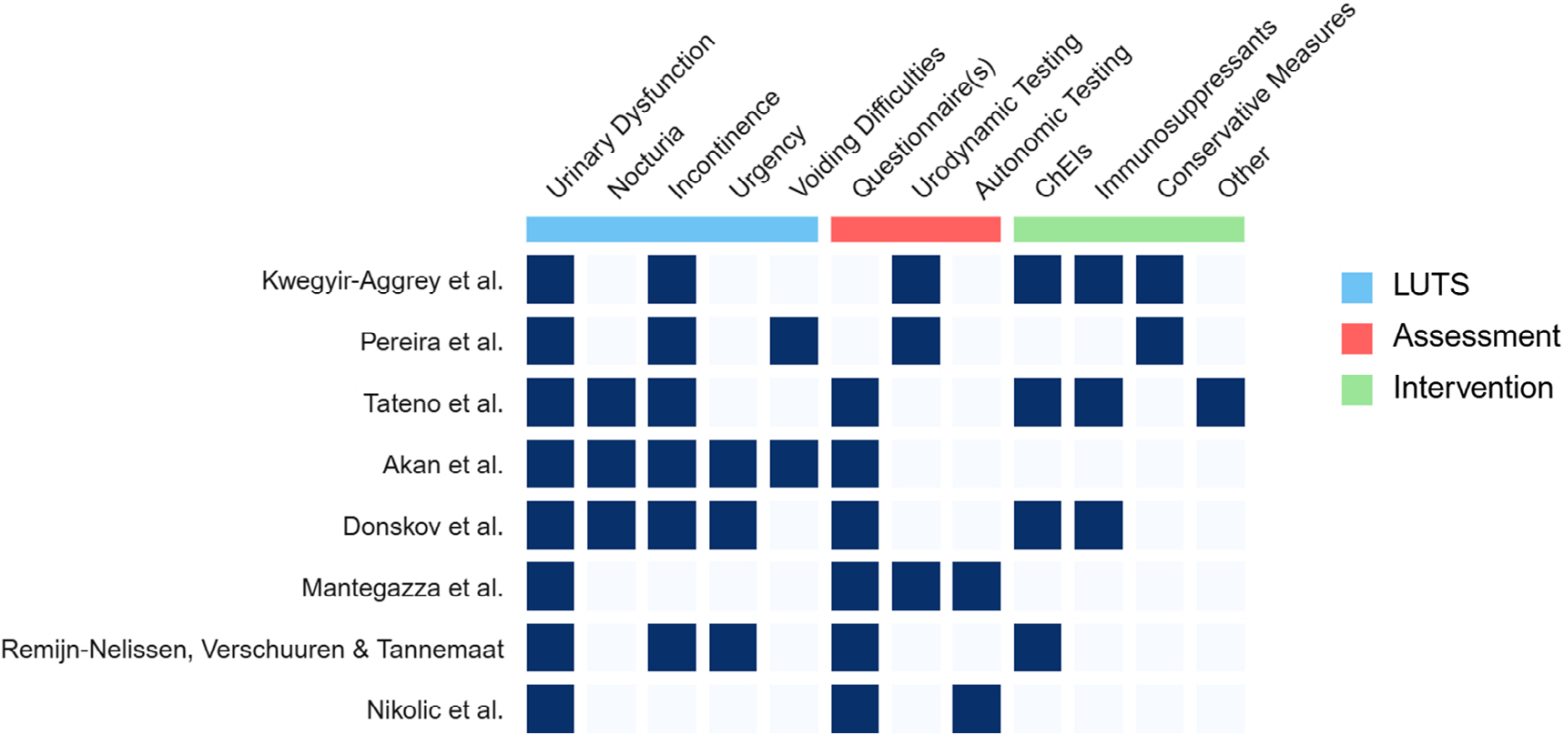
Overview of reported urinary symptoms, assessment methods, and treatment‐related associations across included studies. The figure summarizes the reported lower urinary tract symptoms (blue), urinary symptom assessment methods (red), and interventions or treatment‐related associations (green) across the included studies. It provides a visual overview of the heterogeneity of reported clinical features, diagnostic approaches, and management‐related observations in myasthenic syndromes. Created with BioRender.

Across the included MG studies, urinary symptoms were reported, although frequencies varied markedly according to study design, symptom definition, and patient subgroup (Table 2; Figure 2). In the three case–control studies, storage LUTS predominated: urgency, incontinence, and nocturia were reported more frequently in patients with MG than in healthy controls [19, 20], and overall overactive bladder and bother scores were higher in MG even where symptom‐specific frequencies were not reported (Table 2) [21]. In the prospective study by Nikolic et al., urinary dysfunction was most frequent in AChR‐positive MG with thymoma and least frequent in MuSK‐positive MG, with marked subgroup differences (Table 2) [24]. In contrast, voiding and bladder‐emptying abnormalities were described less consistently and were mainly documented in individual case reports or urodynamic assessments, including residual urine, reduced sphincter contractility, intrinsic sphincter dysfunction, and detrusor underactivity [17, 18]. Two case reports described stress incontinence as the primary presenting urinary symptom in MG, with exertion‐related worsening in one case [17, 18]. Associations between urinary symptoms and disease‐related factors were inconsistent across studies: no consistent relationship was observed with QMG score or MGFA classification, whereas findings regarding antibody status and MG subgroup were heterogeneous [19, 20, 24]. By contrast, treatment‐related associations were more consistent. One case–control study reported higher OAB scores at higher pyridostigmine doses, suggesting a dose‐related association [21], and Remijn‐Nelissen et al. reported more frequent urinary urgency among current pyridostigmine users than among untreated or previously treated patients, although the underlying values were derived from a graphical presentation (Table 2) [23]. Tateno et al. also noted more frequent urgency and urinary frequency in patients receiving cholinesterase inhibitors, although this did not reach statistical significance [19]. In LEMS, one registry‐based study reported abnormal bladder function in the context of autonomic involvement, but no more detailed urinary phenotyping was provided (Table 2) [22]. No eligible studies addressing urinary dysfunction in CMS were identified.

**TABLE 2:** Study‐level quantitative urinary findings in myasthenic syndromes.

Disease	Study	Population	Quantitative urinary finding	Comparator/subgroup finding	Key note
MG	Tateno et al. [19]	*n* = 21	Urgency 7/21 (33.3%); incontinence 9/21 (42.9%); nocturia 5/21 (24.0%)	Controls: urgency 17.2%; incontinence 21%; nocturia 5.6%	All more frequent in MG
MG	Akan et al. [20]	*n* = 36	Nocturia 29/36 (80.5%); incontinence 22/36 (61%); urgency 17/36 (47%); residual urine 9/36 (25.0%); voiding symptoms 3/36 (8.3%)	Higher symptom burden and OABSS vs. controls	Nocturia more common in LOMG
MG	Donskov et al. [21]	*n* = 83	Symptom‐specific frequencies not reported	Higher total OAB and bother scores vs. controls	Higher OAB scores with pyridostigmine doses > 300 mg/day
MG	Remijn‐Nelissen, Verschuuren & Tannemaat [23]	*n* = 410	Urinary urgency approximately 35% in current pyridostigmine users	Approximately 12% in untreated/previously treated patients	Derived from graphical presentation
MG	Nikolic et al. [24]	*n* = 71* (AChR+ thymoma *n* = 21; AChR+ without thymoma *n* = 23; MuSK+ *n* = 23)	Urinary dysfunction: AChR+ thymoma 17/21 (81.0%); AChR+ without thymoma 6/23 (26%); MuSK+ 5/23 (22%)	Marked subgroup differences	Highest frequency in AChR+ thymoma
LEMS	Mantegazza et al. [22]	*n* = 69	Abnormal bladder function 24%	No control group	Recorded within autonomic dysfunction profile

*Note:* This table summarizes study‐level quantitative data on urinary symptoms from the included observational and case–control studies. Case reports are described in Table 1 and the main text but were not included in this overview because they do not permit comparison across cohorts. Frequencies are reported as given in the original studies or calculated from reported numerators and denominators where possible.

Abbreviations: AChR, acetylcholine receptor; LEMS, Lambert‐Eaton myasthenic syndrome; LOMG, late‐onset myasthenia gravis; LUTS, lower urinary tract symptoms; MG, myasthenia gravis; MuSK, muscle‐specific kinase; OAB, overactive bladder; OABSS, Overactive Bladder Symptom Score.

### 3.4 Sex‐Specific Differences in Urinary Symptoms

Sex‐specific analyses were limited and yielded inconsistent findings. Tateno et al. reported that urinary urgency appeared more pronounced in men with MG, whereas stress incontinence within the MG group was observed only in women, although these differences did not reach statistical significance [19]. Akan et al. found no significant effect of sex on urinary symptom scores, uroflowmetry, or ultrasonographic parameters in MG [20]. Remijn‐Nelissen et al. similarly noted that urinary urgency was reported more often in men, but without a significant sex difference [23]. Overall, the currently available evidence does not support consistent sex‐specific patterns of urinary dysfunction in myasthenic syndromes.

### 3.5 Quality of Life

Urinary dysfunction was associated with impaired quality of life in both MG case reports, with patients describing significant distress and limitations in daily activities [17, 18]. In one case–control study, approximately one‐third of MG patients identified bladder symptoms as contributing to reduced quality of life [19], while another study reported higher total bother scores among patients with urinary symptoms compared to those without [20].

### 3.6 Treatment

Management of urinary symptoms in MG was variably reported and not systematically evaluated across studies. Treatment‐related associations primarily involved cholinesterase inhibitors (ChEIs), particularly pyridostigmine, which were consistently associated with urgency, incontinence, and overactive bladder–like symptoms. A dose‐related association was reported, with a higher symptom burden at higher pyridostigmine doses [21]. Consistently, one study reported more frequent urinary urgency among patients receiving pyridostigmine than among untreated patients [23]. Evidence for therapeutic interventions for LUTS was limited. Immunosuppressive therapy and pyridostigmine were not associated with improvement in urinary symptoms, whereas complete resolution following rituximab (RTX) was reported in a single case [17]. Symptom improvement with milnacipran was described in one patient [19]. Conservative measures, including incontinence pads and pelvic floor exercises, were ineffective in two cases [17, 18]. No studies systematically evaluated standard urological or pharmacological treatments for LUTS. In LEMS, registry data reported urinary symptoms in a subset of patients but did not provide information on treatment response, including to amifampridine or pyridostigmine [22].

## 4 Discussion

This scoping review found that urinary dysfunction has been reported in myasthenic syndromes, but the available evidence is limited, heterogeneous, and largely based on small observational studies. Most data derive from MG, whereas evidence for LEMS is sparse, and no eligible studies were identified for CMS. Across the included studies, the reported symptom profile was dominated by storage LUTS, particularly urgency, nocturia, increased frequency, pollakiuria, and urinary incontinence, while voiding and bladder‐emptying abnormalities were described less consistently. Assessment methods varied substantially and included patient‐reported questionnaires, clinical examination, urodynamic testing, imaging, and autonomic assessments. Reported treatment‐related associations were largely observational and most consistently implicated ChEIs, particularly pyridostigmine, in symptom worsening.

Proposed mechanisms include pelvic floor muscle weakness, autonomic involvement, and treatment‐related cholinergic effects [14]. In MG, isolated urodynamic findings such as reduced sphincter contractility and intrinsic sphincter deficiency support a possible contribution of striated pelvic floor weakness [17, 18, 19, 25], but urinary symptoms were not consistently associated with QMG scores, MGFA classification, or antibody status [19, 20]. Autonomic mechanisms are also plausible, particularly given the recognized autonomic involvement in LEMS and reports of urgency and overactive bladder‐like symptoms in MG [24, 26, 27, 28, 29]. However, urinary dysfunction in LEMS has been described only in a single registry study without detailed phenotyping [22]. Treatment‐related effects likely also contribute, as pyridostigmine and other ChEIs may stimulate muscarinic receptors in the lower urinary tract and thereby promote urgency, incontinence, and overactive bladder‐like symptoms [21, 23, 30]. This interpretation is supported by dose‐dependent associations between pyridostigmine exposure and urinary symptom burden [21, 23], although these findings remain observational and do not establish causality. Evidence for treatment of urinary symptoms was sparse and largely limited to individual reports describing pelvic floor exercises [17], incontinence pads [18], milnacipran [19], or immunomodulatory interventions [19, 22, 23, 24], with inconsistent outcomes.

The clinical significance of these findings should be interpreted cautiously. LUTS are highly prevalent in the general population [31, 32, 33, 34, 35, 36, 37], and only a minority of the included studies incorporated control groups. Even in case–control studies [19, 20, 21], controls were small and not always matched for important confounders, including age, sex, body mass index, and comorbidities. Accordingly, the extent to which reported urinary symptoms reflect disease‐related manifestations rather than background prevalence remains uncertain. The available data therefore support urinary dysfunction as a potentially relevant but incompletely characterized feature of myasthenic syndromes rather than an established disease‐specific manifestation.

### 4.1 Limitations

This review is limited by the small and heterogeneous evidence base, comprising only eight studies with predominantly observational designs and small sample sizes [17, 18, 19, 20, 21, 24]. Assessment methods varied substantially across studies; urinary symptoms were captured with different instruments, some of them non‐validated [19], and objective urological evaluations were inconsistently applied or absent [17, 18, 19, 20, 21, 24], increasing the likelihood of underreporting and misclassification. The available literature was also unevenly distributed across disease subtypes, with most data derived from MG, only a single registry‐based study in LEMS [22], and no eligible studies in CMS. In addition, treatment exposure and other clinical variables were not reported consistently, limiting comparison across studies and interpretation of symptom associations. Some reported symptoms also warrant cautious interpretation; polyuria, for example, reflects increased urine production rather than a primary bladder storage or voiding disorder and may therefore indicate systemic or metabolic disease rather than lower urinary tract dysfunction [38].

### 4.2 Conclusions

Taken together, the current literature suggests that urinary dysfunction may occur in myasthenic syndromes and may contribute to reduced quality of life, but the evidence base remains insufficient to define its prevalence, clinical significance, or underlying mechanisms with confidence. More systematic evaluation using validated urinary symptom instruments and standardized objective assessments is needed. Prospective studies with appropriate control groups will be essential to distinguish disease‐related urinary dysfunction from background prevalence and treatment‐related effects, and to guide evidence‐based management strategies.

## Author Contributions

**Rafael Meleka Hanna:** writing – original draft, visualization. **Pratishtha P. Wadnerkar:** writing – review and editing, methodology, validation. **Julia M. Augustin:** writing – original draft, writing – review and editing, conceptualization, investigation. **Blanca G. Sánchez Navarro:** methodology, visualization, writing – review and editing, writing – original draft, data curation, validation. **Franz Blaes:** writing – review and editing, writing – original draft, methodology. **Atay Vural:** writing – review and editing, methodology. **Holly M. Nelson:** writing – review and editing, writing – original draft, visualization, validation. **Avan Kader:** writing – review and editing, methodology, validation. **Hellen Schmidt:** methodology, writing – original draft, software, data curation. **Ayşe N. Özdağ Acarlı:** writing – review and editing, methodology. **Piraye Oflazer:** writing – review and editing, methodology. **Lillith Horstkorte:** methodology, writing – review and editing, writing – original draft, visualization, formal analysis, investigation, validation, software, data curation. **Inga Koneczny:** writing – review and editing, methodology, validation. **Miriam L. Fichtner:** supervision, resources, project administration, writing – review and editing, writing – original draft, methodology, conceptualization.

## Disclosure

We confirm that we have read the Journal's position on issues involved in ethical publication and affirm that this report is consistent with those guidelines.

## Conflicts of Interest

The authors declare no conflicts of interest.

## Supporting information


**Figure S1:** Risk‐of‐bias assessment of included studies using ROBINS‐I. Risk of bias was assessed across the following domains: bias due to confounding (D1), selection of participants (D2), classification of interventions (D3), deviations from intended interventions (D4), missing data (D5), measurement of outcomes (D6), and selection of the reported result (D7). Domain‐level judgments are shown as low, moderate, or serious risk of bias. Created using robvis.

## Data Availability

The data that support the findings of this study are available from the corresponding author upon reasonable request.
